# Research productivity during orthopedic surgery residency correlates with pre-planned and protected research time: a survey of German-speaking countries

**DOI:** 10.1007/s00167-020-05983-w

**Published:** 2020-04-17

**Authors:** Andreas Voss, Björn Andreß, Leo Pauzenberger, Elmar Herbst, Jonas Pogorzelski, Dominik John, Daniel Smolen, Philip P. Roessler, Daniel G. Tobert, Jakob T. Sieker, For the Society of Arthroscopy and Joint Surgery (AGA) Residents Forum, Research Working Group

**Affiliations:** 1 Department of Trauma Surgery, University Medical Center Regensburg, Franz-Josef-Strauß-Allee 11, 93053 Regensburg, Germany; 2 Sporthopaedicum Straubing-Regensburg, Regensburg, Germany; 3 Department of Orthopaedic Sports Medicine, Technical University of Munich, Munich, Germany; 4 Department of Orthopedic Surgery, Catholic Clinics Koblenz-Montabaur, Koblenz, Germany; 5 Sports Surgery Clinic, Dublin, Ireland; 6 Department of Trauma, Hand and Reconstructive Surgery, University Hospital Münster, Münster, Germany; 7 West German Knee and Shoulder Center, Cologne, Germany; 8 Shoulder and Elbow Department, Alphaclinic, Zurich, Switzerland; 9 Ludwig Boltzmann Institute (LBI) for Experimental and Clinical Traumatology, Vienna, Austria; 10 Department of Orthopedics and Trauma Surgery, University Hospital Bonn, Bonn, Germany; 11 Department of Orthopedic Surgery, Massachusetts General Hospital, Boston, MA USA; 12 Department of Orthopedic Surgery, Boston Children’s Hospital, Harvard Medical School, Boston, MA USA; 13 Department of Pathology and Laboratory Medicine, Tufts Medical Center, Tufts University School of Medicine, Boston, MA USA

**Keywords:** Research, Orthopedic surgery training, Traumatology training, Residency

## Abstract

**Purpose:**

The purpose of this study was to identify modifiable factors associated with research activity among residents working in orthopedic surgery and traumatology.

**Methods:**

Residents at 796 university-affiliated hospitals in Austria, Germany, and Switzerland were invited to participate. The online survey consisted of questions that ascertained 13 modifiable and 17 non-modifiable factors associated with the residents’ current research activities. Responses of 129 residents were analyzed. Univariate linear regression was used to determine the association of individual factors with the current research activity (hours per week). The impact of significant non-modifiable factors (with unadjusted *p* values < 0.05) was controlled for using multivariate linear regression.

**Results:**

The univariate analysis demonstrated six non-modifiable factors that were significantly associated with the current research activity: a University hospital setting (*p* < 0.001), an A-level hospital setting (*p* = 0.024), Swiss residents (*p* = 0.0012), the completion of a dedicated research year (*p* = 0.007), female gender (*p* = 0.016), and the department’s size (*p* = 0.048). Multivariate regression demonstrated that the number of protected research days per year (*p* < 0.029) and the percentage of protected days, that were known 1 week before (*p* < 0.001) or the day before (*p* < 0.001), were significantly associated with a higher research activity.

**Conclusions:**

As hypothesized, more frequent and predictable protected research days were associated with higher research activity among residents in orthopedic surgery and traumatology.

**Level of evidence:**

III.

**Electronic supplementary material:**

The online version of this article (10.1007/s00167-020-05983-w) contains supplementary material, which is available to authorized users.

## Introduction

Motivating and enabling residents to perform research during residency training is a challenge for program directors and department chairs [9]. The implementation of changes to resident curricula requires careful consideration from the program leadership. Often, objective data regarding the potential ramifications of these changes are unavailable. In particular, there is a lack of knowledge on the consequences of such changes. Often, these changes are implemented on a trial and error basis.

Additionally, new working regulations within the European Union have forced employers to reduce, adapt, and control the workload of residents to 50 h per week (in many countries even 40 h per week). A reduced workload was enacted to enable residents with time for independent study, research and establish a healthy work–life balance. A similar regulation has been set in the United States in 2002 [7]. The clinical workload was reduced to maximum of 80 h per week. Analysis regarding resident’s authorships significantly increased compared to the years before the workload regulation [7]. Also, the level of evidence of resident-authored papers has improved. Basic science papers were more likely to have a resident first author, indicating that clinical workload reduction had a positive influence on research output.

A study by Williams et al. [11] could demonstrate a significantly higher research output in American orthopedics residency programs with protected research time compared to programs without such dedicated time. This protected research time has often been touted as a solution to motivate trainee research. However, the addition of protected time for research necessarily decreases vital clinical time and experiences.

The aim of the current study was to identify modifiable factors that are associated with the current research activity of residents in orthopedic surgery and traumatology training programs across Europe. It was hypothesized that higher resident research activity is associated with the presence of faculty members who produce high-volume, meaningful research, the presence of robust research infrastructure and funding, as well as dedicated time for research.

## Materials and methods

### Study design

A cross-sectional, observational study was performed among residents enrolled in orthopedic surgery and traumatology residency programs across Austria, Switzerland and Germany during 2017. Using an online questionnaire, the resident physicians self-reported current research activity in hours per week, as well as responses for 13 modifiable and 17 non-modifiable factors (see Supplementary Table 1).

### Sample characteristics

An online invitation for participation in the survey was sent to the chairs or program directors of all 796 orthopedic surgery and traumatology programs with university-affiliation across Austria, Germany and Switzerland. Those were subsequently asked to distribute the website address to the online questionnaire amongst their residents. All responses were recorded anonymously. A total of 146 residents completed the survey. Individuals older than 40 years (*N* = 13) of age were excluded, as they were deemed to not reflect the typical age range of orthopedic residents, which could influence their research activity (e.g. medicine as second career, family responsibilities). Individuals with more than 40 h per week of research (*N* = 4) were excluded as these respondents were presumed to be currently engaged in full-time research. Therefore, the responses of 129 residents were available for analysis.

When a resident responded that the longest duration of their training covered by one employment contract was 0 month then this entry was replaced by a missing value (*N* = 28). Contracts are expected to be at least 1 month of duration and, therefore, a response of 0 month was assumed to be erroneous. If respondents entered a percentage of protected days plannable the week before that was greater than the percentage plannable at least the day before, both entries were replaced by a missing value (*N* = 5), as at least one of the values was assumed to be erroneous. One response (*N* = 1) indicated that their institution was privately as well as government/community owned which was considered mutually exclusive and, therefore, both entries were replaced by a missing value. If a respondent indicated the faculty to resident ratio was ≤ 0 or ≥ 5 then the entries for department size, program size and faculty to resident ratio were replaced by missing values (*N* = 14). The faculty to resident ratio was calculated based on reported information on department size (that is number of residents and faculty combined) and program size (that is number of residents only). The number of faculty was calculated by subtracting the program size from the department size. If a respondent provided a department size smaller than the program size the calculated number of faculty and resulting faculty to resident ratio was less than 0, which is implausible and must represent an erroneous response. Conversely, an example for a faculty to resident ratio greater than 5 would be a program with 5 residents and 25 faculty members. Although the cut-off of 5 is somewhat arbitrary, a faculty ratio of 5 or higher was deemed to be unlikely and possibly caused by an erroneous response. If a resident completed full-time research (i.e. a fellowship, research leave) of ≥ 1 year duration and of ≥ 3 to < 12 month duration, then the response for the ≥ 3 to < 12 months research leave were replaced by missing values (*N* = 13). This was done so that the effects of the longer research leave were not attributed to the shorter leave in the statistical analysis.

The respondents included in the analysis had an age of 30.9 ± 2.8 years (mean ± standard deviation) and completed 3.5 ± 2.0 years of residency training. Residents enrolled in German programs constituted 64% of the sample, while residents enrolled in Swiss and Austrian programs constituted 22% and 14%, respectively. The mean current research activity was 5.2 ± 7.4 h/week (detailed sample characteristics are available in Table 1; sample characteristics by country are provided in Supplementary Table 2).

**Table 1:** Sample characteristics

Characteristic	Sample^§^	*N*
Dependent variable		
Current research activity: h/week	5.2 ± 7.42	128
Modifiable factors		
Faculty research output: Likert scale 1–5	3, 1–5	128
Faculty research quality: Likert scale 1–5	3, 1–5	129
Availability of research infrastructure: Likert scale 1–5	3, 1–5	128
Availability of research-related knowledge: Likert scale 1–5	2, 1–5	129
Availability of research funding: Likert scale 1–5	3, 1–5	129
Availability of salary funding: Likert scale 1–5	2, 1–5	129
Maximum duration of single employment contract: months	31.81 ± 20.42	94
Protected days: days/year	5.41 ± 16.32	127
Predictability of protected days on the prior day: %	8.11 ± 21.71	118
Predictability of protected days in the prior week: %	6.62 ± 19.04	120
Support of ≥ 3 to < 12 months leave: Likert scale 1–5	2, 1–5	127
Support of ≥ 1 year leave, not counted towards training: Likert scale 1–5	2, 1–5	129
Support of ≥ 1 year leave, counted towards training: Likert scale 1–5	2, 1–5	129
Non-modifiable factors		
Completed years of training: years	3.5 ± 2.02	126
Age: years	30.94 ± 2.82	125
Female gender: no. (%)	41, (32%)	127
Completion of ≥ 1 year leave: no. (%)	14, (11%)	128
Completion of ≥ 3 to < 12 months leave: no. (%)	16, (14%)	115
Employment in Austria: no. (%)	18, (14%)	129
Employment in Germany: no. (%)	82, (64%)	129
Employment in Switzerland: no. (%)	29, (22%)	129
Employment in A-level hospital: no. (%)	24, (19%)	129
Employment in B-level hospital: no. (%)	9, (7%)	129
Employment in a University hospital: no. (%)	40, (31%)	129
Employment in Maximum care hospital: no. (%)	19, (15%)	129
Employment in a University teaching affiliate: no. (%)	15, (12%)	129
Employment in privately held institution: no. (%)	19, (37%)	52
Department size: no. of faculty and residents	32.31 ± 18.28	111
Program size: no. of residents	17.5 ± 11.43	110
Faculty to resident ratio	1.05 ± 0.74	108

^§^Mean ± SD for continuous variables; median, range for ordinally scaled variables and number, (% of *N*) for binary variable

### Statistical analyses

Univariate linear regression was used to determine the association of each of the 30 individual factors (13 modifiable and 17 non-modifiable factors listed in Supplementary Table 1) with the current research activity. The non-modifiable risk factors (*p* < 0.05) identified by the univariate analysis were included in a multivariate regression model to control for these factors. Bonferroni adjustment calculations were performed to account for repeated tests (i.e. for testing 13 modifiable and 17 non-modifiable factors). Associations with *p* values < 0.05 were considered significant. To gauge feasibility, an a priori power analysis was performed and a sample size of 53 was determined sufficient to yield a power of 0.80 to detect associations with *R*^2^ values of 0.25 at an *α* error probability of 0.0017 (corresponding to 0.05 adjusted for multiple tests of 13 modifiable and 17 non-modifiable factors). The power analysis was performed using G*Power 3.1 [4]. All other analyses were performed using R version 3.3.2 (Copyright 2016, The R Foundation, Vienna, Austria).

## Results

**Fig. 1:**
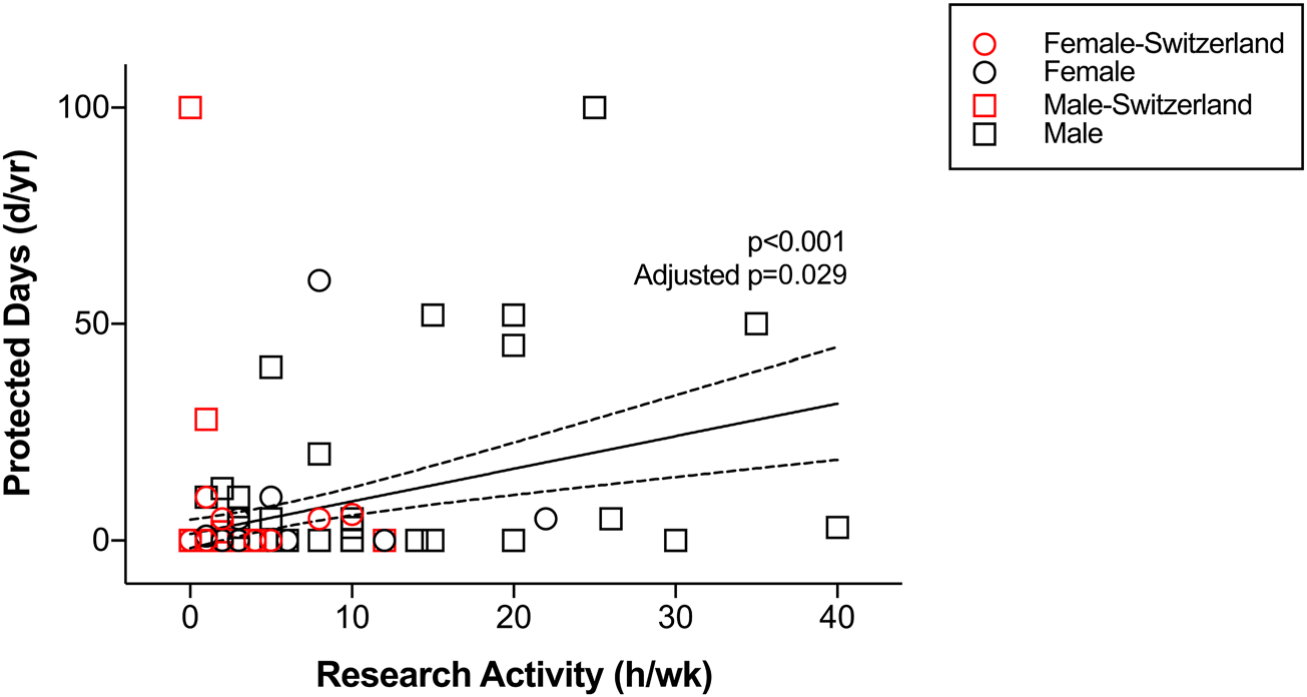
Association of protected days with resident physician’s current research activity

**Fig. 2:**
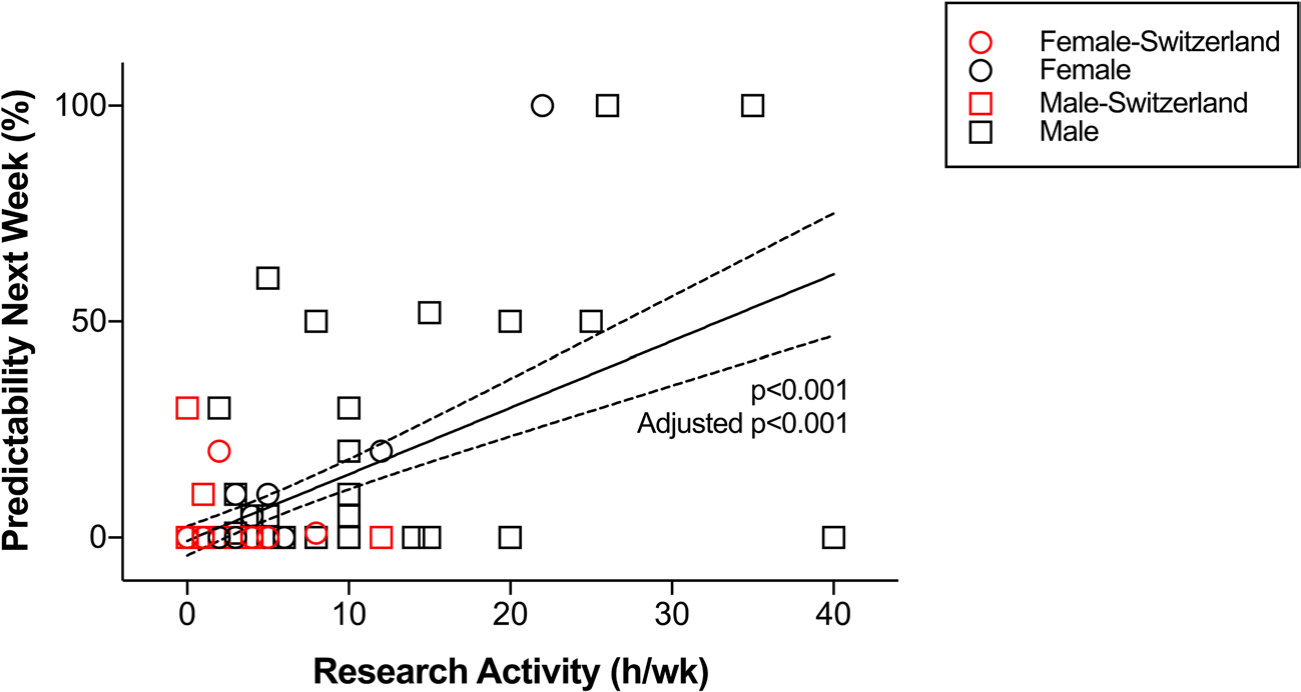
Association of predictability of protected days in the prior week with resident physician’s current research activity

Sample characteristics are displayed in Table 1. The univariate analysis identified protected research days (*p* < 0.001), the predictability of protected days in the prior week (*p* < 0.001), the predictability of protected days 1 day previously (*p* < 0.001), the faculty research output (*p* < 0.001) and quality (*p* < 0.001), the availability of research infrastructure (*p* < 0.001), the support of a ≥ 1 year research time (*p* = 0.005), and the support of a 3–12 month research time (*p* = 0.010) as significantly associated with increased current research activity. Of the non-modifiable factors, the univariate analysis identified employment in a university hospital (*p* < 0.001), completion of ≥ 1-year time for research purposes (*p* = 0.007), and department size (*p* = 0.048) as factors associated with increased current research activity. In contrast, employment in Switzerland (*p* = 0.012), female gender (*p* = 0.016), and employment in an A-level hospital (*p* = 0.024) were negatively associated with research activity (Figs. 1, 2; Table 2).

**Table 2:** Association with current research activity

	Univariate analysis			Multivariate analysis^§^			
	*N*	Coefficient (95% CI)	*p*	*N*	Coefficient (95% CI)	*p*	Adjusted *p*
Modifiable factors							
Predictability of protected days in the prior week: %	120	0.22 (0.16, 0.27)	< 0.001	105	0.21 (0.14, 0.28)	< 0.001	< 0.001
Predictability of protected days on the prior day: %	118	0.18 (0.13, 0.23)	< 0.001	103	0.16 (0.1, 0.23)	< 0.001	< 0.001
Protected days: days/year	127	0.16 (0.08, 0.23)	< 0.001	110	0.13 (0.06, 0.21)	< 0.001	0.029
Faculty research output: Likert scale 1–5	127	1.93 (0.97, 2.9)	< 0.001	109	1.58 (0.43, 2.74)	0.009	n.s.
Faculty research quality: Likert scale 1–5	128	1.80 (0.83, 2.76)	< 0.001	110	1.76 (0.6, 2.91)	0.004	n.s.
Availability of research infrastructure: Likert scale 1–5	127	1.78 (0.79, 2.76)	< 0.001	109	1.53 (0.29, 2.77)	0.017	n.s.
Support of ≥ 1 year leave, not counted towards training: Likert scale 1–5	128	1.45 (0.46, 2.44)	0.005	110	0.42 (− 0.81, 1.64)	n.s.	n.s.
Support of ≥ 3 to < 12 months leave: Likert scale 1–5	126	1.31 (0.33, 2.29)	0.010	108	0.88 (− 0.35, 2.12)	n.s.	n.s.
Support of ≥ 1 year leave, counted towards training: Likert scale 1–5	128	0.81 (− 0.37, 1.99)	n.s.	110	0.57 (− 0.86, 1.99)	n.s.	n.s.
Maximum duration of single employment contract: months	94	− 0.03 (− 0.11, 0.05)	n.s.	84	− 0.05 (− 0.14, 0.03)	n.s.	n.s.
Availability of research-related knowledge: Likert scale 1–5	128	0.23 (− 0.85, 1.31)	n.s.	110	0.02 (− 1.29, 1.33)	n.s.	n.s.
Availability of salary funding: Likert scale 1–5	128	− 0.23 (− 1.4, 0.94)	n.s.	110	− 0.66 (− 1.93, 0.6)	n.s.	n.s.
Availability of research funding: Likert scale 1–5	128	0.07 (− 1.06, 1.2)	n.s.	110	− 0.42 (− 1.73, 0.89)	n.s.	n.s.
Non-modifiable factors							
Employment in a University hospital: yes	128	5.46 (2.85, 8.08)	< 0.001	110	3.03 (− 0.25, 6.32)	n.s.	n.s.
Completion of ≥ 1 year leave: yes	127	5.62 (1.59, 9.65)	0.007	110	4.41 (0.25, 8.58)	0.040	n.s.
Employment in Switzerland: yes	128	− 3.91 (− 6.91, − 0.9)	0.012	110	− 3.33 (− 9.27, 2.61)	n.s.	n.s.
Female gender: yes	127	− 3.37 (− 6.08, − 0.65)	0.016	110	− 2.82 (− 5.78, 0.15)	n.s.	n.s.
Employment in A-level hospital: yes	128	− 3.78 (− 7.02, − 0.54)	0.024	110	− 0.71 (− 7.14, 5.72)	n.s.	n.s.
Department size: no. of faculty and residents	111	0.08 (0, 0.16)	0.048	110	0.07 (− 0.01, 0.15)	n.s.	n.s.
Employment in Germany: yes	128	2.44 (− 0.21, 5.1)	n.s.	110	− 0.47 (− 4.62, 3.68)	n.s.	n.s.
Completion of ≥ 3 to < 12 months leave: yes	114	3.18 (− 0.3, 6.66)	n.s.	96	3.44 (− 0.29, 7.16)	n.s.	n.s.
Program size: no. of residents	110	0.11 (− 0.01, 0.24)	n.s.	108	0.08 (− 0.18, 0.34)	n.s.	n.s.
Employment in B-level hospital: yes	128	− 3.56 (− 8.56, 1.45)	n.s.	110	− 0.51 (− 9.28, 8.26)	n.s.	n.s.
Age: years	125	− 0.25 (− 0.71, 0.22)	n.s.	109	− 0.33 (− 0.83, 0.16)	n.s.	n.s.
Employment in a University teaching affiliate: yes	128	2.04 (− 1.95, 6.04)	n.s.	110	3.65 (− 0.86, 8.16)	n.s.	n.s.
Employment in Maximum care hospital: yes	128	− 1.84 (− 5.45, 1.78)	n.s.	110	− 3.40 (− 7.26, 0.46)	n.s.	n.s.
Faculty to resident ratio	108	0.74 (− 1.26, 2.75)	n.s.	107	0.48 (− 1.38, 2.34)	n.s.	n.s.
Employment in Austria: yes	128	1.06 (− 2.73, 4.86)	n.s.	110	0.47 (− 3.68, 4.62)	n.s.	n.s.
Completed years of training: years	126	− 0.16 (− 0.81, 0.49)	n.s.	109	− 0.23 (− 0.91, 0.45)	n.s.	n.s.
Employment in privately held institution: yes	51	− 0.47 (− 2.78, 1.85)	n.s.	41	− 0.72 (− 3.81, 2.38)	n.s.	n.s.

*n.s.* not significant

^§^Adjusted for employment in a University hospital, completion of ≥ 1 year leave, employment in Switzerland, female gender, employment in A-level hospital, and department size

After adjusting for the employment in a university-affiliated hospital, completion of ≥ 1 year research time, employment in Switzerland, female gender, employment in an A-level hospital, and department size, as well as a subsequent Bonferroni correction of the *p* values for 30 tested factors, the multivariate regression analysis identified protected research days (*p* = 0.029), the predictability of protected days in the prior week (*p* < 0.001), and the predictability of protected days on the prior day (*p < *0.001) as significantly positively associated with increased current research activity (Table 2).

## Discussion

The most important finding of this study was that, as hypothesized, protected research time is associated with a higher self-reported research activity of orthopedic surgery and traumatology residents. Furthermore, the current study demonstrated that advance knowledge of protected research time is positively associated with research activity. This is relevant for programs that have protected research time built into the curriculum as advance notifications are a relatively low-resource change for programs. This study did not confirm the hypotheses that the presence of faculty members who produce high-volume, meaningful research and the presence of robust research infrastructure and funding are associated with the residents’ research activity.

In the univariate analysis, this study further highlights that research activity is influenced by factors that cannot be changed by the program leadership, such as the geography, type of hospital, and other intrinsic factors to the program. Adjusted for such factors, in the multivariate analysis, one additional protected day per week is estimated to increase research activity by ~ 7 h per week (95% confidence interval of ~ 3 to ~ 11 h per week). Prior work of others supports that the available time is the major factor determining research activity. Al-Taha et al. [2] showed that the top perceived barrier to conducting research was the lack of time (83%) amongst Canadian plastic surgery residents participating in a national multicenter cross-sectional study. Levy et al. [7] showed an increase in publications by non-board certified surgeons, as well as an increase in resident first-authorship following the institution of the 80-h workweek limit in 2002. Williams et al. [11] came to the same conclusion. In contrast to these findings, Krueger et al. [6] investigated three different orthopedic residency programs: (1) had a mandatory research year for all residents, (2) had an elective research year for one resident, (3) had no dedicated research time for residents. Interestingly, the research programs with dedicated research time did not produce significantly more or higher quality publications. The authors concluded that research output may be more related to staff engagement, intrinsic motivation for research, and the availability of mentorship programs for the residents. These findings also confirm the results from Osborn et al. [8] with no difference in research output and dedicated research time, but a significantly higher output by faculty with more than 10 years of experience. Of note, Levy et al. [7] and Williams et al. [11] had more residents included in their study compared to Krueger et al. [6], which may indicate a lack of power in the latter study.

A more concerning publication by Grova et al. [5] found a significant decline of self-reported surgical skills and clinical aptitude in residents who spent a year or longer of dedicated research time. They concluded that their findings may have a dramatic effect on the confidence of residents in their further clinical career. This has also been shown by D’Angelo et al. [3], who proposed that the use of simulation might maintain procedural skills and confidence in residents pursuing dedicated research time. Thus, when implementing strategies to motivate residents for research, it is important to implement measures to maintain clinical and surgical skills. This may implicate a structured research program to make sure that clinical performance will not suffer from dedicated research time.

In addition to providing more protected time, the current study highlights the importance of advance scheduling. The study indicates that if scheduling can be improved such that a resident has advance knowledge (> 1 week) of a research day for half, instead of a quarter of these occurrences the resident’s research activity increases by approximately 5 h per week (~ 4 to ~ 7 h per week). This increase is without providing any additional protected days. Although not significant after adjusting for multiple tests, residents who completed a dedicated research year are estimated to perform approximately 4 h more research per week (~ 0–9 h per week) than residents who did not. While there may be an effect of a dedicated research year on subsequent research activity, this study suggests that providing plannable protected days throughout the residency training is more strongly related to resident’s research activity and may be a more effective measure than supporting a dedicated research year.

After lack of time, the second most commonly perceived barrier to research amongst the plastic surgery residents surveyed by Al-Taha et al. [2] was a lack of research supervisors and mentors. Though not significant in our study after controlling for non-modifiable factors and adjusting for multiple tests, there may be an association between the faculty’s research output and quality and the resident’s research activity. Residents’ who agree with the statement that the attending surgeons at their institution perform high-quality research are estimated to engage in approximately 4 h (~ 1– 6 h) more research per week than residents who disagree. Sufficiently powered additional research is needed to confirm this association. A complex research ethics/institutional review process has been previously described as a frequently perceived barrier [2], but was not included in our questionnaire. Developing a faculty with high research quality and output that can provide supervision and mentoring may be an effective measure to increase resident physicians’ research activity.

While this study focuses on the identification of modifiable factors associated with residents’ research activity, this study further highlights that research activity may be influenced by factors that cannot be changed by the program leadership. Of note, in the univariate analysis, residents in Switzerland are estimated to spend ~ 4 h less on research per week (95% confidence interval of ~ 1– 7 h per week) than residents outside of Switzerland. However, this finding is not statistically significant after correcting for the testing of multiple hypotheses and sufficiently powered additional research is needed to confirm this association. Swiss programs did not differ significantly in the predictability and number of protected days, and their residents even reported a somewhat increased perceived availability of research-related knowledge, funding, and support to spend ≥ 1 year of dedicated research time (Supplementary Table 2). Notably, the maximum duration of employment contracts are estimated to be of ~ 11 months shorter duration in Swiss programs (~ 1– 20 months) for an average of 23 months (Supplementary Table 2). However, the maximum employment contract duration was not significantly associated with the residents’ research activity (Table 2). This current study did not identify organizational differences or other factors that could explain a potentially reduced research activity amongst residents in Swiss programs.

In addition to the factors included in this study, orthopedic and trauma surgery residency programs are also affected by broad societal and generational changes [10]. These changes may also be important determinants of research interest and productivity during residency training.

The study sample only represents a small fraction of the orthopedic surgery and traumatology residents across Austria, Switzerland, and Germany. Although the total number of residents is not publicly available, we identified 796 programs with university-hospital affiliation that we have contacted and received responses from 129 residents that were included in the analysis. The inability to contact residents directly likely contributed to the relatively low response rate. Thus, our findings might not be applicable to all programs, but should apply to all programs that disseminated our invitation to their residents. The latter programs are considered much more likely to make use of the reported data, which mitigates this limitation to some degree. The relatively low response rate could also be due to a low interest amongst residents in pursuing research. The availability of pertinent data is limited, but Ahn et al. found amongst U.S. orthopedic surgery residents that interest is quite high, with only 30% of residents being uninterested in performing research. In addition, this rate was not different between survey responders and non-responders [1]. However, data on research interest amongst European orthopedic surgery residents are not available, and it would be prudent to assume that respondents to our survey had a higher than average interest in performing research. Thus, our findings may only apply to programs with residents that show a prior interest in performing research. The study design, including the adjustment for substantial non-modifiable factors and a strict correction for multiple hypothesis testing, was primarily geared towards identifying those modifiable factors with a strong association to the resident’s research activity. It is, however, limited in demonstrating the absence of an association and factors without significant association may still have a substantial effect. Furthermore, the self-reported time spent on research in hours per week as a dependent variable has been chosen. While hours per week is a meaningful variable to gauge a resident’s current research activity, it is not a direct measure of research output. As it does provide a more granular metric for research activity compared to the number of publications, given that the time spent on each publication can vary widely, it was selected as most suitable measure for this investigation. Information on whether and to which extent research activity is performed during as opposed to outside of regular working hours was not obtained in this study but may be an important factor associated with residents’ research activity.

## Conclusion

As hypothesized, protected research days and, perhaps more importantly, predictable protected research days are significantly associated with more research activity in orthopedic surgery and traumatology resident physicians. In contrast, hypotheses that the presence of faculty members who produce high-volume, meaningful research and the presence of robust research infrastructure and funding are associated with the residents’ research activity were not confirmed. Therefore, program directors and department chairs seeking to promote research activity should focus on creating plannable protected time for research.

## Electronic supplementary material

Below is the link to the electronic supplementary material.

Supplementary file 1 (XLSX 12 kb)

Supplementary file 2 (DOCX 104 kb)
