# Supplementary material for: Research productivity during orthopedic surgery residency correlates with pre-planned and protected research time: a survey of German-speaking countries
Source: Knee Surg Sports Traumatol Arthrosc. 2020 Apr 17;29(1):292–9. doi: 10.1007/s00167-020-05983-w (PMC7862526; doi:10.1007/s00167-020-05983-w)
Supplement: Supplementary file 2 — (DOCX 104 kb) [file 167_2020_5983_MOESM2_ESM.docx]

**Supplementary Table 2.** Sample Characteristics by Country

|  | *Austria* | | *Germany* | | *Switzerland* | | *Switzerland vs. Non-Switzerland* |
| --- | --- | --- | --- | --- | --- | --- | --- |
| *Characteristic* | *Sample ^§^* | *N* | *Sample ^§^* | *N* | *Sample ^§^* | *N* | *p ^§§^* |
|  |  |  |  |  |  |  |  |
| **Dependent Variable** |  |  |  |  |  |  |  |
| Current research activity *– hours/week* | 6.12 ± 5.93 | 17 | 6.07 ± 8.47 | 82 | 2.17 ± 3.08 | 29 | 0.012 |
|  |  |  |  |  |  |  |  |
| **Modifiable Factors** |  |  |  |  |  |  |  |
| Faculty research output *– Likert scale 1–5* | 3, 1–5 | 18 | 2, 1–5 | 82 | 3, 1–5 | 28 | n.s. |
| Faculty research quality *– Likert scale 1–5* | 4, 1–5 | 18 | 3, 1–5 | 82 | 3, 1–5 | 29 | n.s. |
| Availability of research infrastructure *– Likert scale 1–5* | 3, 2–5 | 18 | 2, 1–5 | 82 | 3, 1–5 | 28 | n.s. |
| Availability of research-related knowledge *– Likert scale 1–5* | 3, 1–5 | 18 | 2, 1–5 | 82 | 4, 1–5 | 29 | 0.002 |
| Availability of research funding *– Likert scale 1–5* | 3, 1–5 | 18 | 2, 1–5 | 82 | 3, 1–5 | 29 | 0.006 |
| Availability of salary funding *– Likert scale 1–5* | 3, 1–5 | 18 | 2, 1–5 | 82 | 3, 1–4 | 29 | n.s. |
| Maximum duration of single employment contract *– months* | 27.17 ± 21.95 | 12 | 35.56 ± 21.1 | 62 | 22.95 ± 13.77 | 20 | 0.028 |
| Protected days *– days/year* | 3.56 ± 12.98 | 16 | 5.78 ± 16.04 | 82 | 5.38 ± 19.03 | 29 | n.s. |
| Predictability of protected days on the prior day*– %* | 1.25 ± 3.87 | 16 | 10.75 ± 25.6 | 75 | 4.85 ± 13.68 | 27 | n.s. |
| Predictability of protected days in the prior week *– %* | 0.94 ± 2.72 | 16 | 9.32 ± 22.99 | 77 | 2.26 ± 6.97 | 27 | n.s. |
| Support of ≥3 to <12 months leave *– Likert scale 1–5* | 3, 1–5 | 18 | 2, 1–5 | 80 | 3, 1–5 | 29 | n.s. |
| Support of ≥1 year leave, not counted towards training *– Likert scale 1–5* | 2.5, 1–5 | 18 | 2, 1–5 | 82 | 2, 1–4 | 29 | n.s. |
| Support of ≥1 year leave, counted towards training *– Likert scale 1–5* | 2, 1–4 | 18 | 2, 1–5 | 82 | 2, 1–5 | 29 | 0.004 |
|  |  |  |  |  |  |  |  |
| **Non-modifiable Factors** |  |  |  |  |  |  |  |
| Completed years of training *– yr* | 3.31 ± 1.62 | 16 | 3.6 ± 2.08 | 82 | 3.32 ± 2.11 | 28 | n.s. |
| Age *– yr* | 31.25 ± 3.53 | 16 | 31.06 ± 2.67 | 80 | 30.41 ± 2.86 | 29 | n.s. |
| Female gender *– no. (%)* | 4, (25%) | 16 | 27, (33%) | 82 | 10, (34%) | 29 | n.s. |
| Completion of ≥1 year leave *– no. (%)* | 2, (11%) | 18 | 7, (9%) | 81 | 5, (17%) | 29 | n.s. |
| Completion of ≥3 to <12 months leave *– no. (%)* | 3, (19%) | 16 | 8, (11%) | 75 | 5, (21%) | 24 | n.s. |
| Employment in Austria *– no. (%)* | 18, (100%) | 18 | 0, (0%) | 82 | 0, (0%) | 29 | 0.014 |
| Employment in Germany *– no. (%)* | 0, (0%) | 18 | 82, (100%) | 82 | 0, (0%) | 29 | <0.001 |
| Employment in Switzerland *– no. (%)* | 0, (0%) | 18 | 0, (0%) | 82 | 29, (100%) | 29 | - |
| Employment in A-level hospital *– no. (%)* | 2, (11%) | 18 | 0, (0%) | 82 | 22, (76%) | 29 | <0.001 |
| Employment in B-level hospital *– no. (%)* | 0, (0%) | 18 | 1, (1%) | 82 | 8, (28%) | 29 | <0.001 |
| Employment in a University hospital *– no. (%)* | 5, (28%) | 18 | 35, (43%) | 82 | 0, (0%) | 29 | <0.001 |
| Employment in Maximum care hospital *– no. (%)* | 5, (28%) | 18 | 14, (17%) | 82 | 0, (0%) | 29 | 0.011 |
| Employment in a University teaching affiliate *– no. (%)* | 3, (17%) | 18 | 12, (15%) | 82 | 0, (0%) | 29 | 0.027 |
| Employment in privately held institution *– no. (%)* | 0, (0%) | 5 | 17, (45%) | 38 | 2, (22%) | 9 | n.s. |
| Department size *– no. of faculty and residents* | 29 ± 15.93 | 14 | 31.26 ± 19.76 | 74 | 37.7 ± 13.61 | 23 | n.s. |
| Program size *– no. of residents* | 12.23 ± 7.03 | 13 | 17.61 ± 12.8 | 74 | 20.13 ± 7.25 | 23 | n.s. |
| Faculty to resident ratio | 1.86 ± 1 | 13 | 0.95 ± 0.68 | 73 | 0.88 ± 0.4 | 22 | n.s. |

n.s. = not significant

^§^ Mean ± SD for continuous variables; Median, Range for ordinally scaled variables and number, (% of *N*) for binary variable

^§§^ Unadjusted P-value of univariate linear regression
